# Trends in the incidence and associated factors of late-onset sepsis associated with improved survival in extremely preterm infants born at 23–26 weeks’ gestation: a retrospective study

**DOI:** 10.1186/s12887-018-1130-y

**Published:** 2018-05-23

**Authors:** Jin Kyu Kim, Yun Sil Chang, Sein Sung, So Yoon Ahn, Won Soon Park

**Affiliations:** 1 Department of Pediatrics, Chonbuk National University School of Medicine, Jeonju, 54907 Korea; 2 Department of Pediatrics, Samsung Medical Center, Sungkyunkwan University School of Medicine, Seoul, 06351 Korea; 3 Research Institute of Clinical Medicine of Chonbuk National University-Biomedical Research Institute of Chonbuk National University Hospital, Jeonju, 54907 Korea

**Keywords:** Extremely preterm infants, Late-onset sepsis, Associated factor, Survival rate

## Abstract

**Background:**

To investigate the trends in the incidence and associated factors of late-onset sepsis (LOS) associated with improved survival in extremely preterm infants.

**Methods:**

Medical records of 364 infants who were born at 23–26 weeks’ gestation from 2000 to 2005 (period I, *n* = 124) and from 2006 to 2011 (period II, *n* = 240) were retrospectively reviewed. The infants were stratified into subgroups of 23–24 and 25–26 weeks’ gestation within each period, and survival, LOS rate, and clinical characteristics were analyzed. Multivariate logistic regression analyses were completed to identify the clinical factors associated with LOS.

**Results:**

The survival rate of 75.8% during period I significantly improved to 85.4% during period II, especially in infants at 23–24 weeks’ gestation (55.1% vs. 78.1%, respectively). The LOS rate of 33.1% during period I significantly reduced to 15.8% during period II, especially in infants at 25–26 weeks’ gestation (32.0% vs. 8.9%, respectively). The LOS rate per 1000 hospital days of 4.0 during period I significantly reduced to 1.8 during period II. *Candida* presence reduced from 21.3% during period I to 4.7% during period II. In multivariate analyses, during period I, prolonged intubation, especially in infants at 25–26 weeks’ gestation, and necrotizing enterocolitis, especially in infants at 23–24 weeks’ gestation, were significantly associated with LOS.

**Conclusions:**

Improved survival of infants at 23–24 weeks’ gestation was associated with a simultaneous reduction of LOS incidence in infants at 25–26 weeks’ gestation. Less-invasive assisted ventilation may be one of the details of improved perinatal and neonatal care that has contributed to lowering risk of infection or death among periviable infants.

**Electronic supplementary material:**

The online version of this article (10.1186/s12887-018-1130-y) contains supplementary material, which is available to authorized users.

## Background

The risk of late onset sepsis (LOS) increases with decreasing birth weight and gestational age, likely due to the more immature immune function in these infants, the increased use of invasive devices including endotracheal intubation and vascular catheterization, and prolonged hospital stay [1–3]. Therefore, the actual number of infants at a high risk for developing LOS might be increasing with the current improved survival of extremely low birth weight infants (ELBWI) [4–8]. Although with recent improvements in neonatal intensive care medicine, reports from come multicenter consortia indicate some morbidities, including late onset infection, have been reduced in frequency, they still persist, and continue to be a significant cause of morbidities and mortality [9–13].

The variable incidence of LOS found among neonatal intensive care units (NICUs) [14, 15] suggests that besides the immaturity of these infants, environmental factors in the NICU, such as clinical practice style and management decisions, might also contribute to the risk of LOS [6, 14]. Previously, we observed that the implementation of certain clinical strategies, including the restrictive use of antibiotics; less use of invasive procedures, such as umbilical vessel catheterization and endotracheal intubation; and the establishment and strict application of guidelines for hand washing, gloving, infant handling, and central intravascular line management, was quite effective in reducing the incidence of LOS in ELBWI [16]. Taken together, these findings suggest that the development of new and effective therapeutic strategies could reduce the incidence of LOS and improve the clinical outcomes of ELBWI [15, 17–20].

Recently, we noted markedly improved survival rates with improvements in perinatal and neonatal intensive care in extremely preterm (EPT) infants near the limit of viability at 23–26 weeks’ gestation [12, 13]. Therefore, in the present retrospective observational study, we investigated whether improved survival in EPT infants at 23–26 weeks’ gestation was associated with an altered incidence of LOS and, if applicable, the clinical factors related to this alteration.

## Methods

The medical records of infants born at 23–26 weeks’ gestation and who were admitted into the Samsung Medical Center NICU from January 1, 2000 to December 31, 2005 (period I, *n* = 124) and January 1, 2006 to December 31, 2011 (period II, *n* = 240) were retrospectively reviewed. All of the infants had at least more than one blood culture. The study periods were divided according to the improved survival rate of these EPT infants, and the infants were stratified into 23–24 and 25–26 weeks’ gestation subgroups within each period.

Maternal and neonatal characteristics, including mortality and various major morbidities, as well as the incidence of LOS during admission, were assessed in the 23–24 and 25–26 weeks’ gestation subgroups during the study periods. Gestational age was determined through best obstetric estimates and the modified Ballard test. Survival rate was assessed until discharge from the NICU. An episode of LOS was defined as positive blood cultures in symptomatic patients after 72 h of life with concurrent use of antibiotics for more than 5 days, or those treated for a shorter period if the patient died. If the same organism was cultured after 15 days of appropriate antibiotic therapy, or if a different organism was cultured from a subsequent culture after appropriate antibiotic therapy, this was considered an additional episode. The exposure to associated factors was determined from the date of admission into the NICU to the date of discharge or death. The durations of intubation, nasal continuous positive airway pressure (CPAP), percutaneous central venous catheter, and postnatal steroid use before and after positive culture were calculated.

Prolonged rupture of membrane (PROM) was defined as rupture of membranes more than 24 h prior to delivery. Pregnancy-induced hypertension, maternal steroid use, and histologic chorioamnionitis were classified based on maternal hospital records. Outcome measures, including respiratory distress syndrome (RDS), patent ductus arteriosus (PDA), necrotizing enterocolitis (NEC), bronchopulmonary dysplasia (BPD), intraventricular hemorrhage (IVH), and retinopathy of prematurity (ROP), were analyzed. RDS was defined as clinical features of respiratory distress requiring surfactant and ventilator treatment within the first 24 h of life. PDA screening was routinely performed using echocardiography in all infants after 7 days of life. NEC was classified according to the system of Bell et al. [21]. BPD was defined as an oxygen requirement at 36 weeks’ postmenstrual age [22]. IVH was diagnosed using brain ultrasonography and graded according to Papile’s classification system [23]. ROP was categorized according to the international classification of ROP and based on the most advanced ROP stage observed [24]. Appropriate for gestational age (AGA) was defined as birth weight between the 90th and 10th percentile.

### Statistical analysis

All analyses were based on the first episode of LOS. Associations between LOS and maternal and neonatal variables, hospital course, and mortality were explored. Continuous variables are expressed as mean ± standard deviation, while categorical variables are expressed as number and percentage. Comparisons between continuous variables were performed using the Mann–Whitney *U* test or Student’s *t-*test, while comparisons between categorical variables were performed using the chi-squared test or Fisher’s exact test. Logistic regression analysis was performed to control for all variables and to determine the significant independent factors associated with LOS. Adjusted odds ratios (ORs) and 95% confidence intervals (CIs) were calculated for each possible associated factor. Binary logistic regression was used for unadjusted and adjusted analyses, in which missing values were removed. SPSS version 17 (SPSS Inc., Chicago, IL, USA) was used for all statistical analyses, and *p*-values of < 0.05 were considered statistically significant.

## Results

### Trends in the survival rate and incidence of late-onset sepsis according to period

Table 1 shows the survival rate and incidence of LOS among infants in each subgroup and period. The overall survival rate of 75.8% (94/124) during period I significantly improved to 85.4% (205/240) during period II (*p* = 0.023). In the subgroup analyses, survival rate during period I significantly improved during period II in infants at 23–24 weeks’ gestation (55.1% [27/49] vs. 78.1% [82/105], respectively; *p* = 0.003), but not in infants at 25–26 weeks’ gestation (89.3% [67/75] vs. 91.1% [123/135], respectively; *p* = 0.674).

**Table 1:** Survival and incidence of late-onset sepsis among infants in each subgroup and period

	23–24 weeks’ gestation		25–26 weeks’ gestation		Total, 23–26 weeks’ gestation	
	P I (*n* = 49)	P II (*n* = 105)	P I (*n* = 75)	P II (*n* = 135)	P I (n = 124)	P II (n = 240)
Survival at discharge, n (%)	27 (55.1)	82 (78.1) *	67 (89.3)	123 (91.1)	94 (75.8)	205 (85.4) *
LOS infants, n (%)	17 (34.7)	26 (24.8)	24 (32.0)	12 (8.9) *	41 (33.1)	38 (15.8) *
LOS episodes, n	21	31	26	12 *	47	43 *
LOS rate/1000 hospital days	5.1	2.6 *	3.5	1.0 *	4.0	1.8 *

*P* period, *LOS* late-onset sepsis

*p < 0.05 compared to period I

The overall LOS incidence of 47 episodes in 41/124 (33.1%) infants during period I significantly reduced to 43 episodes in 38/240 (15.8%) infants during period II (*p* < 0.001). In the subgroup analyses, the incidence of LOS during period II was significantly lower compared to that in period I in infants at 25–26 weeks’ gestation only (12 episodes in 12/135 [8.9%] infants vs. 26 episodes in 24/75 [32.0%] infants, respectively; *p* < 0.001); this was not observed in infants at 23–24 weeks’ gestation (31 episodes in 26/105 [24.8%] infants vs. 21 episodes in 17/49 [34.7%] infants, respectively; *p* = 0.201).

The overall LOS rate/1000 hospital days of 4.0 during period I significantly reduced to 1.8 during period II (*p* = 0.002). In the subgroup analyses, the LOS rate/1000 hospital days of 5.1 and 3.5 in infants at 23–24 and 25–26 weeks’ gestation, respectively, during period I significantly reduced to 2.6 and 1.0, respectively, during period II (*p* = 0.017 and *p* = 0.011, respectively).

### Trends in pathogen distribution according to period

Table 2 shows the pathogen distribution among infants with LOS in each subgroup and period. With the reduction of the overall incidence of LOS, the proportionate incidence of *Candida albicans* infection significantly decreased in period II compared to that in period I (*p* < 0.001), particularly in infants at 23–24 weeks’ gestation (*p* = 0.002). The overall proportionate distribution of other pathogenic gram-positive or gram-negative organisms was not significantly different between the two periods.

**Table 2:** Pathogen distribution among infants in each subgroup and period

	23–24 weeks’ gestation		25–26 weeks’ gestation		Total, 23–26 weeks’ gestation	
	Period I (*n* = 21)	Period II (*n* = 31)	Period I (*n* = 26)	Period II (n = 12)	Period I (*n* = 47)	Period II (*n* = 43)
*Gram (+)*	6 (28.5)	19 (61.3) *	18 (69.2)	7 (59.3)	24 (51.5)	26 (60.5)
*CoNS*	2 (9.5)	7 (22.6)	6 (23.1)	3 (25)	8 (17.0)	10 (23.3)
*Staphylococcus aureus*	3 (14.3)	3 (9.7)	6 (23.1)	1 (8.3)	9 (19.1)	4 (9.3)
*Enterococcus*	0 (0)	6 (19.4) *	3 (11.5)	1 (8.3)	3 (6.4)	7 (16.3)
*Streptococcus group B*	1 (4.8)	3 (9.7)	1 (3.8)	1 (8.3)	2 (4.3)	4 (9.3)
Others	0 (0)	0 (0)	2 (7.7)	1 (8.3)	2 (4.3)	1 (2.3)
*Gram (−)*	8 (38.1)	10 (32.3)	5 (19.2)	5 (41.7)	13 (27.7)	15 (34.9)
*Enterobacter*	2 (9.5)	2 (6.5)	1 (3.8)	2 (16.7)	3 (6.4)	4 (9.3)
*Escherichia coli*	0 (0)	2 (6.5)	1 (3.8)	2 (16.7)	1 (2.1)	4 (9.3)
*Klebsiella*	1 (4.8)	2 (6.5)	2 (7.7)	0 (0)	3 (6.4)	2 (4.7)
*Pseudomonas*	3 (14.3)	0 (0) *	0 (0)	0 (0)	3 (6.4)	0 (0)
*Acinetobacter*	1 (4.8)	1 (3.2)	1 (3.8)	0 (0)	2 (4.3)	1 (2.3)
Others	1 (4.8)	3 (9.7)	0 (0)	1 (8.3)	1 (2.1)	4 (9.3)
*Fungus*	7 (33.3)	2 (6.5) *	3 (11.5)	0 (0)	10 (21.3)	2 (4.7) *
*Candida albicans*	6 (28.6)	1 (3.2) *	3 (11.5)	0 (0)	9 (19.1)	1 (2.3) *
*Caddida parapsilosis*	1 (4.8)	1 (3.2)	0 (0)	0 (0)	1 (2.1)	1 (2.3)

Data are presented as number (percentage). Organisms counts differ from the frequency of infants with late-onset sepsis due to multiple episodes. *CoNS, Coagulase Negative Staphylococcus.* * *p* < 0.05 compared to period I

### Trends in clinical characteristics and associated factors of late-onset sepsis according to period

Table 3 shows the clinical characteristics and associated factors of LOS among infants in each subgroup and period. During period II, the overall gestational age and birth weight in infants at 23–26 weeks’ gestation with LOS were significantly lower compared with those of the control group (*p* = 0.003 and *p* = 0.008, respectively). The overall five-minute Apgar scores in infants at 23–26 weeks’ gestation with LOS were significantly lower compared with those of infants in the control group (*p* = 0.002). However, the Apgar scores of both infants with LOS and those in the control group were significantly higher during period II than during period I. The overall durations of intubation, postnatal steroid use, and antibiotics in infants at 23–26 weeks’ gestation with LOS were significantly longer compared with those of the control group, especially in infants at 25–26 weeks’ gestation during period II compared to period I.

**Table 3:** Clinical characteristics and associated factors of late-onset sepsis in each subgroup and period

23–24 weeks’ gestation	Period I (n = 49)		Period II (n = 105)		Total (*n* = 154)	
	LOS (*n* = 17)	Control (*n* = 32)	LOS (n = 26)	Control (*n* = 79)	LOS (n = 43)	Control (*n* = 111)
Gestational age, wk	23.8 ± 0.4	23.7 ± 0.5	23.6 ± 0.5	23.6 ± 0.5	23.7 ± 0.5	23.7 ± 0.5
Body weight, g	644.5 ± 95.5	671.9 ± 124.4	633.8 ± 73.5	639.7 ± 103.4	638.0 ± 82.0	649.0 ± 110.2
One-min Apgar score	2.5 ± 1.3	2.7 ± 1.4	4.3 ± 1.5 *	4.5 ± 1.3 *	3.6 ± 1.7	4.0 ± 1.5
Five-min Apgar score	5.6 ± 1.2	5.6 ± 2.0	7.2 ± 1.3 *	7.1 ± 1.1 *	6.5 ± 1.5	6.7 ± 1.6
AGA	14 (82.4)	28 (87.5)	25 (96.2)	73 (92.4)	39 (90.7)	101 (91.0)
Pathologic CA	8 (47.1)	15 (46.9)	14 (53.8)	44 (55.7)	22 (51.2)	59 (53.2)
Maternal steroid use	11 (64.7)	22 (68.8)	22 (84.6)	66 (83.5)	33 (76.7)	88 (79.3)
Intubation duration, d	51.8 ± 43.2	34.5 ± 24.8	51.2 ± 46.0	41.8 ± 27.2	51.4 ± 44.4	39.7 ± 26.6
N-CPAP duration, d	19.1 ± 35.0	18.5 ± 20.2	34.6 ± 40.5	32.5 ± 23.3 *	28.5 ± 38.7	28.4 ± 23.3
Postnatal steroid use duration, d	14.8 ± 23.6	7.1 ± 8.0	23.8 ± 37.4	15.2 ± 11.6 *	11.2 ± 5.4	12.0 ± 6.5
Umbilical catheter duration, d	8.8 ± 3.9	8.4 ± 7.0	12.8 ± 5.8 *	13.4 ± 5.7 *	11.2 ± 5.4	12.0 ± 6.5
PCVC duration, d	27.1 ± 28.4	23.2 ± 18.0	64.8 ± 64.8 *	53.6 ± 37.1 *	49.9 ± 56.2	44.8 ± 35.5
Antibiotics use duration, d	28.4 ± 26.9	19.6 ± 14.3	39.5 ± 32.0	29.2 ± 23.5 *	35.1 ± 30.2	26.4 ± 21.6
Hospital stay, d	88.4 ± 67.6	82.1 ± 67.1	120.5 ± 92.4	113.3 ± 48.5 *	107.8 ± 84.1	104.3 ± 56.0
25–26 weeks’ gestation	Period I (n = 75)		Period II (n = 135)		Total (*n* = 210)	
	LOS (n = 24)	Control (*n* = 51)	LOS (n = 12)	Control (*n* = 123)	LOS (*n* = 36)	Control (*n* = 174)
Gestational age, wk	25.5 ± 0.5	25.5 ± 0.5	25.2 ± 0.4	25.5 ± 0.5 #	25.4 ± 0.5	25.5 ± 0.5 #
Body weight, g	804.1 ± 139.7	809.6 ± 124.2	759.7 ± 155.3	813.4 ± 121.4	789.3 ± 144.4	812.3 ± 121.9
One-min Apgar score	2.9 ± 1.7	3.6 ± 1.6	5.0 ± 1.8 *	5.1 ± 1.5 *	3.6 ± 2.0	4.7 ± 1.7
Five-min Apgar score	5.7 ± 1.5	6.8 ± 1.4 #	7.0 ± 1.9 *	7.4 ± 1.2 *	6.1 ± 1.8	7.2 ± 1.3
AGA	22 (91.7)	48 (94.1)	9 (75.0)	115 (93.5) #	31 (86.1)	163 (93.7)
Pathologic CA	10 (41.7)	20 (40.0)	5 (41.7)	61 (49.6)	15 (41.7)	81 (46.8)
Maternal steroid use	16 (66.7)	37 (72.5)	10 (83.3)	100 (81.3)	26 (72.2)	137 (78.7)
Intubation duration, d	44.5 ± 51.9	27.7 ± 33.8	38.0 ± 16.1	18.3 ± 17.8 #	42.4 ± 43.1	21.1 ± 23.9 #
N-CPAP duration, d	16.8 ± 14.5	24.5 ± 7.3	38.1 ± 26.8 *	29.2 ± 19.7	23.9 ± 21.6	27.8 ± 19.1
Postnatal steroid use duration, d	9.2 ± 10.3	6.6 ± 12.8	14.2 ± 10.7	5.6 ± 8.0 #	10.8 ± 10.6	5.9 ± 9.7 #
Umbilical catheter duration, d	5.8 ± 4.7	5.8 ± 5.1	10.4 ± 10.0	5.6 ± 4.9	7.3 ± 7.1	5.6 ± 4.9
PCVC duration, d	22.9 ± 15.9	22.4 ± 16.8	55.4 ± 26.0 *	43.2 ± 28.6 *	33.7 ± 24.9	37.1 ± 27.3
Antibiotics use duration, d	27.8 ± 18.1	18.3 ± 15.9 #	33.2 ± 15.6	17.0 ± 13.8 #	29.6 ± 17.3	17.4 ± 14.4
Hospital stay, d	110.8 ± 61.7	94.8 ± 35.0	121.5 ± 53.2	89.7 ± 32.0 #	114.4 ± 58.4	91.2 ± 32.9 #
Total, 23–26 weeks’ gestation	Period I (n = 124)		Period II (n = 240)		Total (*n* = 364)	
	LOS (41)	Control (83)	LOS (38)	Control (202)	LOS (79)	Control (285)
Gestational age, wk	24.8 ± 0.9	24.8 ± 1.0	24.1 ± 0.9 *	24.8 ± 1.0 #	24.4 ± 1.0	24.7 ± 1.0 #
Body weight, g	737.9 ± 145.6	756.5 ± 140.7	673.5 ± 119.8 *	745.5 ± 142.5 #	706.9 ± 136.8	748.7 ± 141.8 #
One-min Apgar score	2.7 ± 1.5	3.2 ± 1.5	4.6 ± 1.6 *	4.9 ± 1.5 *	3.6 ± 1.8	4.4 ± 1.7 #
Five-min Apgar score	5.6 ± 1.4	6.3 ± 1.7 #	7.1 ± 1.5 *	7.3 ± 1.2 *	6.3 ± 1.6	7.0 ± 1.4 #
AGA	36 (87.8)	76 (91.6)	34 (89.5)	188 (93.1)	70 (88.6)	264 (92.6)
Pathologic CA	18 (43.9)	35 (42.7)	19 (50)	105 (52)	37 (46.8)	140 (49.3)
Maternal steroid use	27 (65.9)	59 (71.1)	32 (84.2)	166 (82.2) *	59 (74.7)	225 (78.9)
Intubation duration, d	47.5 ± 48.0	30.3 ± 30.7 #	47.1 ± 39.3	27.5 ± 24.7 #	47.3 ± 43.7	28.3 ± 26.6 #
N-CPAP duration, d	17.8 ± 24.7	22.2 ± 18.6	35.7 ± 36.4 *	30.5 ± 21.2 *	26.4 ± 32.0	28.1 ± 20.8
Postnatal steroid use duration, d	11.5 ± 17.1	6.7 ± 11.2	20.8 ± 31.6	9.4 ± 10.6	16.0 ± 25.4	8.5 ± 10.8 #
Umbilical catheter duration, d	7.0 ± 4.6	6.8 ± 6.0	12.0 ± 7.3 *	8.6 ± 6.5 #*	9.4 ± 6.5	8.1 ± 6.4
PCVC duration, d	24.6 ± 21.7	22.7 ± 17.2	61.8 ± 55.3 *	47.2 ± 32.5 *	42.5 ± 45.2	40.1 ± 30.9
Antibiotics use duration, d	28.1 ± 21.9	18.8 ± 15.2 #	37.5 ± 27.8	21.8 ± 19.1 #	32.6 ± 25.2	20.9 ± 18.0 #
Hospital stay, d	101.5 ± 64.3	89.9 ± 49.9	120.8 ± 81.3	99.0 ± 40.8	110.8 ± 73.1	96.3 ± 43.7

Data are presented as mean ± standard deviation or number (percentage)

*LOS* late-onset sepsis, *AGA* appropriate for gestational age, *CA* chorioamnionitis, *N-CPAP* nasal continuous positive pressure, *PCVC* percutaneous central venous catheter

# *p* < 0.05 compared to LOS; * *p* < 0.05 compared to period I

### Trends in mortality and morbidities of late-onset sepsis according to period

Table 4 shows the mortality and morbidities of LOS among infants in each subgroup and period. The overall mortality rate in infants at 23–26 weeks’ gestation with LOS was significantly higher compared with that of the control group (*p* = 0.002). In the subgroup analysis, the mortality rate of LOS in infants at 23–24 weeks’ gestation was significantly higher than that of infants in the control group during period II (*p* < 0.001), but not during period I. However, the mortality rate of infants at 23–24 weeks’ gestation in the control group significantly improved during period II compared to during period I (*p* = 0.003). Additionally, the overall incidences of morbidities, such as PDA, NEC, and ROP, in infants at 23–26 weeks’ gestation with LOS were significantly higher compared with those in infants in the control group during period II, but not during period I.

**Table 4:** Mortality and morbidities of late-onset sepsis in each subgroup and period

23–24 weeks’ gestation	Period I (n = 49)		Period II (n = 105)		Total (n = 154)	
	LOS (n = 17)	Control (n = 32)	LOS (n = 26)	Control (n = 79)	LOS (n = 43)	Control (n = 111)
Mortality	9 (52.9)	13 (40.6)	10 (38.5)	13 (16.5) #,*	19 (44.2)	26 (23.4) #
PDA	17 (100)	29 (90.6)	26 (100)	75 (94.8)	43 (100)	104 (93.7)
NEC ≥ stage 2b	3 (17.6)	2 (6.3)	7 (26.9)	7 (8.9) #	10 (23.3)	9 (8.1) #
BPD grade (≥ moderate)	11 (100)	15 (75.0) #	12 (63.2) *	49 (70.0)	23 (76.7)	64 (71.1)
IVH ≥ stage 3	4 (23.5)	11 (34.4)	11 (42.3)	28 (35.4)	15 (34.9)	39 (35.1)
ROP ≥ stage 3	8 (50.0)	11 (36.7)	12 (52.2)	33 (43.4)	20 (51.3)	44 (41.5)
25–26 weeks’ gestation	Period I (n = 75)		Period II (n = 135)		Total (n = 210)	
	LOS (n = 24)	Control (n = 51)	LOS (n = 12)	Control (n = 123)	LOS (n = 36)	Control (n = 174)
Mortality	3 (12.5)	5 (9.8)	1 (8.3)	11 (8.9)	4 (11.1)	16 (9.2)
PDA	23 (95.8)	48 (94.1)	11 (91.7)	105 (85.4)	34 (94.9)	153 (87.9)
NEC ≥ stage 2b	1 (4.2)	2 (3.9)	2 (16.7)	6 (4.9)	3 (8.3)	8 (4.6)
BPD grade (≥ moderate)	12 (54.5)	32 (68.1)	9 (81.8)	47 (42.0) #,*	21 (63.6)	79 (49.7)
IVH ≥ stage 3	3 (12.5)	5 (9.8)	1 (8.3)	16 (13.0)	4 (11.1)	12 (12.1)
ROP ≥ stage 3	11 (47.8)	21 (42.0)	6 (54.5)	27 (23.1) #,*	17 (50)	48 (28.7) #
Total, 23–26 weeks’ gestation	Period I (n = 124)		Period II (n = 240)		Total (n = 364)	
	LOS (*n* = 41)	Control (*n* = 83)	LOS (*n* = 38)	Control (*n* = 202)	LOS (n = 79)	Control (*n* = 285)
Mortality	12 (29.3)	18 (21.7)	11 (28.9)	24 (11.9) #*	23 (29.1)	42 (14.7) #
PDA	40 (97.6)	77 (92.8)	37 (97.4)	180 (89.1)	77 (97.5)	257 (90.2) #
NEC ≥ stage 2b	4 (9.8)	4 (4.8)	9 (23.7)	13 (6.4) #	13 (16.5)	17 (6.0) #
BPD grade (≥ moderate)	23 (69.7)	47 (70.1)	21 (70)	96 (52.7) *	44 (69.8)	143 (57.3)
IVH ≥ stage 3	7 (17.1)	16 (19.3)	12 (31.6)	44 (21.8)	19 (24.1)	60 (21.1)
ROP ≥ stage 3	19 (48.7)	32 (40)	18 (52.9)	60 (31.1) #	37 (50.7)	92 (33.7) #

Data are presented as number (percentage)

*LOS* late-onset sepsis, *PDA* patent ductus arteriosus, *NEC* necrotizing enterocolitis, *BPD* bronchopulmonary dysplasia, *IVH* intraventricular hemorrhage, *ROP* retinopathy of prematurity

# *p* < 0.05 compared to LOS; * *p* < 0.05 compared to period I

### Associated factors for late-onset sepsis

The univariate and multivariate analyses of possible associated factors for LOS are shown in Tables 5 and 6, respectively. While study period; gestational age; birth weight; Apgar score at 5 min; durations of hospitalization, intubation, postnatal steroid use, and antibiotics use; mortality, and incidence of NEC and ROP were significantly associated with LOS in the univariate analysis, only study period, duration of intubation (especially in infants at 25–26 weeks’ gestation), and NEC incidence (especially in infants at 23–24 weeks’ gestation) were significantly associated with LOS in the multivariate analysis.

**Table 5:** Unadjusted univariate analysis of associated factors for late-onset sepsis

Associated factors	23–24 weeks’ gestation	*p*-value	25–26 weeks’ gestation	*p*-value	23–26 weeks’ gestation	*p*-value
	OR(95% CI) (n = 154)		OR(95% CI) (n = 210)		OR(95% CI) (n = 364)	
Period II	0.620(0.297–1.294)	0.203	**0.207(0.096–0.446)**	**< 0.001**	**0.381(0.229–0.634)**	**< 0.001**
Gestational age, wk	1.036(0.489–2.193)	0.927	0.592(0.282–1.243)	0.166	**0.721(0.562–0.924)**	**0.010**
Body weight, 100 g	0.901(0.639–1.270)	0.552	0.871(0.663–1.143)	0.320	**0.813(0.681–0.970)**	**0.021**
Apgar score, 5 min	0.939(0.742–1.187)	0.598	**0.709(0.571–0.880)**	**0.002**	**0.811(0.694–0.947)**	**0.008**
Hospital stay, wk	1.006(0.969–1.044)	0.764	**1.099(1.032–1.170)**	**0.003**	**1.036(1.003–1.070)**	**0.031**
Intubation duration, wk	1.074(0.998–1.155)	0.055	**1.168(1.050–1.299)**	**0.004**	**1.122(1.060–1.187)**	**< 0.001**
Postnatal steroid use, wk	1.132(0.991–1.293)	0.067	**1.322(1.049–1.666)**	**0.018**	**1.215(1.074–1.376)**	**0.002**
Antibiotic use, wk	1.100(0.999–1.212)	0.053	**1.355(1.164–1.578)**	**< 0.001**	**1.191(1.097–1.292)**	**< 0.001**
Mortality	**2.588(1.299–5.452)**	**0.012**	1.234(0.387–3.936)	0.722	**2.376(1.323–4.268)**	**0.004**
NEC, ≥ stage 2b	**3.434(1.286–9.172)**	**0.014**	1.886(0.475–7.487)	0.367	**3.105(1.437–6.711)**	**0.004**
ROP, ≥ stage 3	1.483(0.710–3.100)	0.295	**2.479(1.170–5.255)**	**0.018**	**2.022(1.199–3.411)**	**0.008**

Bolded values indicate significant associated factors at *p* < 0.05

*OR* odds ratio, *CI* confidence interval, *NEC* necrotizing enterocolitis, *ROP* retinopathy of prematurity

**Table 6:** Multivariate logistic regression analysis of associated factors for late-onset sepsis

Associated factors	23–24 weeks’ gestation	*p*-value	25–26 weeks’ gestation	*p*-value	23–26 weeks’ gestation	*p*-value
	OR(95% CI) (n = 154)		OR(95% CI) (n = 210)		OR(95% CI) (n = 364)	
Period II	0.546(0.253–1.179)	0.123	**0.239(0.108–0.530)**	**< 0.001**	**0.346(0.212–0.623)**	**< 0.001**
Intubation duration, wk	**1.077(1.001–1.159)**	**0.047**	**1.133(1.020–1.258)**	**0.020**	**1.115(1.054–1.181)**	**< 0.001**
NEC, ≥ stage 2b	**3.628(1.332–9.883)**	**0.012**	2.344(0.550–9.995)	0.250	**3.376(1.510–7.548)**	**0.003**

Bolded values indicate significant associated factors at *p* < 0.05

OR, odds ratio; CI, confidence interval; NEC, necrotizing enterocolitis

## Discussion

In the present study, the survival rate of EPT infants born at 23–26 weeks’ gestation significantly improved during period II compared to during period I, especially in infants born at 23–24 weeks’ gestation. This improved survival of EPT infants was simultaneously associated with significantly reduced incidence of LOS during period II compared to period I, especially in infants born at 25–26 weeks’ gestation. In concordance with our data, Stoll et al. [25] reported a marked improvement in survival rates during the last decade among infants born between 23 and 24 weeks’ gestation, as well as significantly reduced incidence of LOS and increased intact survival without major morbidities, especially in infants born between 25 and 28 weeks’ gestation. Furthermore, although the plateau in the survival rate of infants born at 25–26 weeks’ gestation has already been reached, improved survival in infants born at 23–24 weeks’ gestation was associated with significantly reduced incidence of BPD in infants born at 25–26 weeks’ gestation [13, 14]. Taken together, these findings suggest that better perinatal and neonatal care practices, which contributed to improved survival of the most immature infants born at 23–24 weeks’ gestation during period II, simultaneously paralleled with the contributions to intact survival without major morbidities in the more mature EPT infants born at 25–26 weeks’ gestation.

The Neonatal Research Network report on improved survival and neurodevelopmental outcome of periviable infants also reported a reduction in LOS, but did not test for the degree of associations between changes in LOS and changes in mortality and improved outcomes [26]. Previously, we observed that improved survival in infants born at 23–24 weeks’ gestation was mainly attributable to significantly reduced proportionate mortality due to sepsis [13]. In the present study, significantly higher mortality was observed in the infants born at 23–24 weeks’ gestation with LOS compared to the control group. However, in the present study, mortality and Apgar score at 5 min, and the clinical factors associated with improved survival that were identified in our previous studies [12, 13] also significantly improved in the control group of these infants during period II compared to during period I, and the association between mortality and LOS shown in the unadjusted univariate analysis in these infants. In addition, routine use of antenatal corticosteroids, which is known to be associated with lower rates of mortality before 25 weeks’ gestation, increased (67 and 84% received antenatal steroids during periods I and II, respectively) [27]. Overall, these findings suggest that improved perinatal and neonatal care rather than reduced incidence of LOS are primarily responsible for the improved survival rates observed during period II in the infants born at 23–24 weeks’ gestation.

It is important to identify the clinical practices primarily responsible for the reduction in LOS in EPT infants. Previously, we observed that the implementation of clinical strategies consisting of the restrictive use of antibiotics; umbilical vessel catheterization and prolonged endotracheal intubation; and the establishment of guidelines for hand washing, gloving, infant handling, and care of central intravascular lines, significantly reduced the rate of LOS in ELBWI [16]. An additional table file shows these clinical practices in more detail (see Additional file 1). In the present study, among the associated factors for LOS, period II; Apgar score at 5 min; and durations of hospitalization, intubation, postnatal steroid use, and antibiotics use were identified as significant using an unadjusted univariate analysis. Only period II and the duration of intubation showed significant association with LOS, especially in infants born at 25–26 weeks’ gestation, in the multivariate logistic regression analysis. These findings suggest that although it might be difficult to specifically identify which of the clinical practices in this intervention strategy actually played an important role, better perinatal and neonatal care practices, including these strategies as a whole, were quite effective in reducing the incidence of LOS, especially in infants born at 25–26 weeks’ gestation during period II.

Endotracheal intubation and prolonged mechanical ventilation are known to significantly increase the risk of LOS due to the breakdown of the physiologic barrier and the colonization of humidified air with hydrophilic bacteria to physical trauma while passing an endotracheal tube and to transient bacteremia during endotracheal suction [28–31]. Therefore, our data on the significant association of LOS with the duration of intubation in the multivariate logistic regression analysis suggests that aggressive early weaning from more invasive intubation and mechanical ventilation to less-invasive assisted ventilation, such as nasal CPAP, is important for reducing the incidence of LOS in EPT infants [16]. Moreover, the duration of intubation and nasal CPAP was significantly associated with the incidence of BPD in EPT infants [12]. In the present study, NEC, especially in infants born at 23–24 weeks’ gestation, was significantly associated with LOS in the multivariate logistic regression analysis. Overall, these findings suggest that the clinical implementation of the less-invasive ventilation strategy is effective for reducing not only LOS, but also other major morbidities associated with extreme prematurity, such as BPD.

Lower gestational age, intravascular catheter use, and prolonged antibiotic therapy are well-known risk factors for *Candida* sepsis [32, 33]. Therefore, our data of reduced proportionate rate of *Candida albicans* sepsis during period II as compared to during period I is difficult to explain, especially in the most immature infants born at 23–24 weeks’ gestation despite significantly prolonged use of central intravascular catheters. At our institution, we did not use fluconazole prophylaxis, but strictly restricted empirical use of broad spectrum antibiotics, including carbapenems, third generation cephalosporins, and vancomycin, only by approval from the pediatric infectious disease division [34]. Period II was the only clinical factor significantly associated with fungal sepsis (OR, 0.165; 95% CI, 0.052–0.521*; p* = 0.002) in the present study. A better infection control strategy, including the establishment of guidelines for central intravascular line management and meticulous aseptic care of the central line during period II, might be responsible for this improvement.

The present study had limitations, including its retrospective and uncontrolled observational study design. Although “study period” was included as an independent factor in the multivariate analysis of associated factors for LOS, there is a possibility that any unknown confounders might not have been controlled for in this retrospective study. Second, we defined that the exposure to associated factors was determined from the date of admission to the date of discharge or death. In this study, it was not apparent whether the associated factors were the cause or result of LOS. The third limitation was that as our data were obtained from a single institution, any positive findings obtained in this study might not be generalizable to another NICU. However, we used a relatively large sample size of 364 EPT infants born between 23 and 26 weeks’ gestation at the same institution with similar baseline clinical characteristics.

Moreover, although the consistency of clinical management was not measured, our data showing more antenatal steroid use and significantly higher one- and five-minute Apgar score during period II in infants of 23–24 weeks’ gestational age suggest that better obstetric perinatal and delivery room managements were provided during period II compared to period I. Thus, these findings imply that this single center study was well-placed in identifying the clinical strategies associated with improved survival and LOS rate, and might also redeem the limitations.

## Conclusions

In summary, better perinatal and neonatal intensive care was likely responsible for improved survival, especially in infants born at 23–24 weeks’ gestation during period II, which was simultaneously paralleled with a reduction in the incidence of LOS, especially in infants born at 25–26 weeks’ gestation. Besides the implementation of clinical strategies for infection control, early extubation and aggressive weaning to less-invasive ventilation techniques, such as nasal CPAP, might be important for these improvements. Our data are in agreement with other studies that show that the development of perinatal and neonatal care improves survival of immature infants and simultaneously reduces morbidities of more mature preterm infants [35, 36]. Collectively, better perinatal and neonatal intensive care could simultaneously lead to improved survival and reduced incidence of LOS in EPT infants.

## Additional files

Additional file 1: **Table S1.** Infection control activities in our NICU. Detailed clinical practices for infection control in our institution’s neonatal intensive care unit. (DOCX 15 kb)

Additional file 2: The datasets supporting the conclusion of this study. (XLSX 112 kb)

## Abbreviations

AGA: Appropriate for gestational age
BPD: Bronchopulmonary dysplasia
CIs: Confidence intervals
CPAP: Continuous positive airway pressure
ELBWI: Extremely low birth weight infants
EPT: Extremely preterm
IVH: Intraventricular hemorrhage
LOS: Late-onset sepsis
NEC: Necrotizing enterocolitis
NICU: Neonatal intensive care unit
ORs: Odds ratios
PDA: Patent ductus arteriosus
PROM: Prolonged rupture of membrane
RDS: Respiratory distress syndrome
ROP: Retinopathy of prematurity
