# Supplementary material for: Trends in the incidence and associated factors of late-onset sepsis associated with improved survival in extremely preterm infants born at 23–26 weeks’ gestation: a retrospective study
Source: BMC Pediatr. 2018 May 23;18:172. doi: 10.1186/s12887-018-1130-y (PMC5966853; doi:10.1186/s12887-018-1130-y)
Supplement: Supplementary file 1 — Table S1. Infection control activities in our NICU. Detailed clinical practices for infection control in our institution’s neonatal intensive care unit. (DOCX 15 kb) [file 12887_2018_1130_MOESM1_ESM.docx]

**Table S1. Infection control activities in our NICU**

| **Infection Control Activity** | |
| --- | --- |
| Infection surveillance | Active surveillance for *S. aureus* and other bacteria/fungi via skin and nasal culture (≥ 1/week) |
|  | Surveillance for VRE, CRE, and respiratory/enteric viruses |
| Infection control monitoring | Regular monitoring by the infection control office  - Hand hygiene  - Aseptic technique during procedure |
|  | Outbreak monitoring and management  - Cohort isolation  - Environment culture (incubator, nursing cart, keyboard, etc.) and environmental disinfection activities |
| Clinical activity | Isolation precaution |
|  | Maximal sterile barrier precautions during central venous catheter insertion (caps and masks, sterile gowns and gloves, large sterile drapes) |
|  | Hand hygiene before and after touching the patient zone |
|  | Hand hygiene plus sterile gloving before and after touching the patient (never touch the baby with bare hands) |
|  | Hand culture feedback |
|  | Antibiotic stewardship (restrictive use of empirical broad spectrum antibiotics) |
| QI activity | Applying VAP prevention bundle |
|  | Applying CLABSI prevention bundle |
| Education | Monthly education for NICU health care providers |
|  | Semi-annual education for hospital health care providers |

NICU, neonatal intensive care unit; *S. aureus, Staphylococcus aureus*; VRE, vancomycin-resistant Enterococci; CRE, carbapenem-resistant Enterobacteriaceae; QI, Quality improvement; VAP, ventilator-associated pneumonia; CLABSI, central line-associated blood stream infection.
